# Lithotomy in a Child Two Years and Eleven Months Old

**Published:** 1849-05

**Authors:** F. H. Hamilton


ART. IV.—*Lithotomy in a Child two years and eleven months old. Patient under the Influence of Chloroform.* By F. H. HAMILTON, M. D.

This lad was born in Pennsylvania, but removed, with his parents, to Rome, Oneida Co., when he was a few months old. About this time, he became troubled with a diarrhœa, which has continued, with only occasional remissions, to the present time. He has had, also, prolapsus ani, and all the usual symptoms of stone in the bladder.

I operated at Rome, Feb. 10th, 1849, in presence of Drs. Bergen, Bassenger, Blair, Pope, and others, by the subpubic, lateralized mode. After the child was placed upon the table, Dr.——, dentist, held a piece of lint, wet with about one drachm of chloroform, to his mouth. In about three minutes, he became quiet. I then introduced the director, at which moment he evacuated his bowels and bladder, and made some complaint. The lint was again held to his mouth, and in one minute he was in a quiet, natural sleep, from which he did not awake until ten minutes after the operation was completed, and after he had been removed to his bed. The operation was made entirely with a common scalpel-shaped bistoury, and occupied, including the removal of the calculus, (an inch and a quarter in diameter) about forty-five seconds. To the perfect quietude of the child, and the use of but one knife throuhgout, I ascribe the rapidity with which I was enabled to make the operation. The child gave no sign of pain during any part of the cutting for, or removal of the stone. No artery was cut, requiring a ligature, the moderate hemorrhage which occurred, being easily controlled with a cone of snow. The calculus was smooth, canullated and ovoid; a phosphate of lime, with lithic acid.

One feature of the operation, I record as novel:—while cutting for the director, I kept the forefinger of my left hand in the rectum, and with the end of the thumb of the same hand, I followed each incision to its bottom; and this I continued to do, until the director was reached. This mode of cutting enables the operator to avoid the rectum in the early part of his incisions.

Two weeks after the operation was made, the wound had healed, and the child was walking about.

